# *ZNF385A* and *ZNF346* Serve as Prognostic Biomarkers Associated with an Inflamed Immunosuppressive Tumor Microenvironment in Hepatocellular Carcinoma

**DOI:** 10.3390/ijms24043155

**Published:** 2023-02-05

**Authors:** Qihang Peng, Jin Li, Qian Wu, Pei Wang, Zhongcui Kang, Yiting Deng, Yu Xiao, Peng Zheng, Feng Ge, Ying Chen

**Affiliations:** 1 College of Life Science, Yangtze University, Jingzhou 434025, China; 2 College of Life Science and Healthy, Wuhan University of Science and Technology, Wuhan 430072, China; 3 State Key Laboratory of Freshwater Ecology and Biotechnology, Institute of Hydrobiology, Chinese Academy of Sciences, Wuhan 430072, China

**Keywords:** *ZNF385A*, *ZNF346*, liver cancer, inflammation, tumor microenvironment, immunotherapy

## Abstract

Hepatocellular carcinoma (HCC) has a high mortality rate worldwide, and there are still many problems in the early diagnosis, molecular targeted therapy, and immunotherapy. It is necessary to explore valuable diagnostic markers and new therapeutic targets in HCC. Zinc finger protein 385A (*ZNF385A*) and zinc finger protein 346 (*ZNF346*) represent a unique class of RNA-binding Cys2 His2 (C2H2) zinc finger proteins that are involved in the regulation of cell cycle and apoptosis, but little is known of their roles in HCC. Based on multiple databases and analysis tools, we explored the expression, clinical relation, prognostic value, possible biological function, and pathways of *ZNF385A* and *ZNF346*, and their relationship with immune infiltration. Our results revealed that *ZNF385A* and *ZNF346* were highly expressed and were associated with poor prognosis in HCC. Hepatitis B virus (HBV) infection may lead to the overexpression of *ZNF385A* and *ZNF346*, which was accompanied by elevated apoptosis and chronic inflammation. Moreover, *ZNF385A* and *ZNF346* were positively correlated with immune-suppressive cells, inflammatory cytokines, immune checkpoint genes, and poor immunotherapy efficacy. Finally, the knockdown of *ZNF385A* and *ZNF346* was observed to negatively affect the proliferation and migration of HepG2 cells in vitro. In conclusion, *ZNF385A* and *ZNF346* are promising candidate biomarkers for the diagnosis, prognosis, and response to immunotherapy in HCC, and this study may help to understand the tumor microenvironment (TME) of liver cancer, and to develop new therapeutic targets.

## 1. Introduction

Primary liver cancer is the third leading cause of cancer deaths worldwide [1]; the five-year relative survival rate in the United States is only 20% [2]. Hepatocellular carcinoma (HCC) is one of the two main histological subtypes (accounting for approximately 90%) [3]. The most common causes of HCC include hepatitis virus (HBV and hepatitis C virus or HCV), metabolic (diabetes and non-alcoholic fatty liver disease or NAFLD), toxic (aflatoxins and alcohol liver diseases or ALD) and immune system-related disorders [4]. Due to very few early symptoms, the diagnosis of HCC is significantly delayed, and it is difficult to receive curative treatments at the earliest opportunity [5]. Over the past decade, molecular targeted therapies have been applied in the treatment of advanced HCC; however, the multikinase vascular endothelial growth factor receptor (VEGFR) inhibitors, including sorafenib and lenvatinib or anti-VEGF/VEGFR monoclonal antibodies, showed limited efficacy and common toxicities [6]. For these reasons, there remains a need for reliable diagnostic markers and an understanding of the molecular mechanisms of hepatocarcinogenesis to develop new therapies.

Recently, immunotherapy has emerged as a revolutionary treatment for cancers. Nevertheless, only a fraction of HCC patients showed a response to immune checkpoint therapy [7]; one possible reason for this is that the immunosuppressive tumor microenvironment (TME) limits the clinical efficacy of immunotherapy [8]. Unfortunately, little is known about how HCC remodels the immunosuppressive TME to in turn evade immune surveillance, as well as to acquire resistance to immune checkpoint inhibitors (ICIs); thus, a better understanding of this process can contribute to predicting and improving the efficacy of immunotherapy.

Cys2 His2 (C2H2) zinc finger proteins (ZNFs) are critical regulators of cancer progression [9]. Although they are most noted for their roles as DNA-binding transcription factors, a few C2H2 ZNFs with RNA binding activity were identified [10]; for instance, *ZNF385A* and *ZNF346*. Both of them have similar zinc finger organization and characteristics, but they are distinctly different from other conventional C2H2 ZNFs, suggesting that they represent a new subfamily [11]. Different studies have revealed the association of *ZNF385A* and *ZNF346* with *P53*, and the two can regulate the expression of *P53* downstream genes involved in cell cycle arrest and apoptosis, indicating their involvement in cell fate determination and cancer initiation. P53 can activate the transcription of *ZNF385A* [12], and ZNF385A can bind with *p53* mRNA or protein to regulate its activity [13]. ZNF346 also directly interacts with P53 protein, and has a regulatory effect [14]. *ZNF346* was reported to contribute to the proliferation and invasion of neuroblastoma [15,16]. Apart from this, their potential roles in cancer development remain largely unknown. This provoked us to explore whether *ZNF385A* and *ZNF346* are involved in cancer initiation and progression.

In this study, we performed a comprehensive analysis of *ZNF385A* and *ZNF346* expression patterns. It was found that elevated *ZNF385A* and *ZNF346* mRNA expression were prognostic predictors of poor overall survival (OS) in patients with HCC. A further exploration of the potential mechanisms of *ZNF385A* and *ZNF346* showed that they may not only be involved in the regulation of tumorigenesis and the development of HCC, but they may also be correlated with an inflammatory immunosuppressive TME and exert a predictive effect on the response to immunotherapy. This study provided preliminary evidence for *ZNF385A* and *ZNF346* as biomarkers and targets in HCC.

## 2. Results

### 2.1. Gene Expression Analysis of ZNF385A and ZNF346 in Pan-Cancer

**Figure 1:**
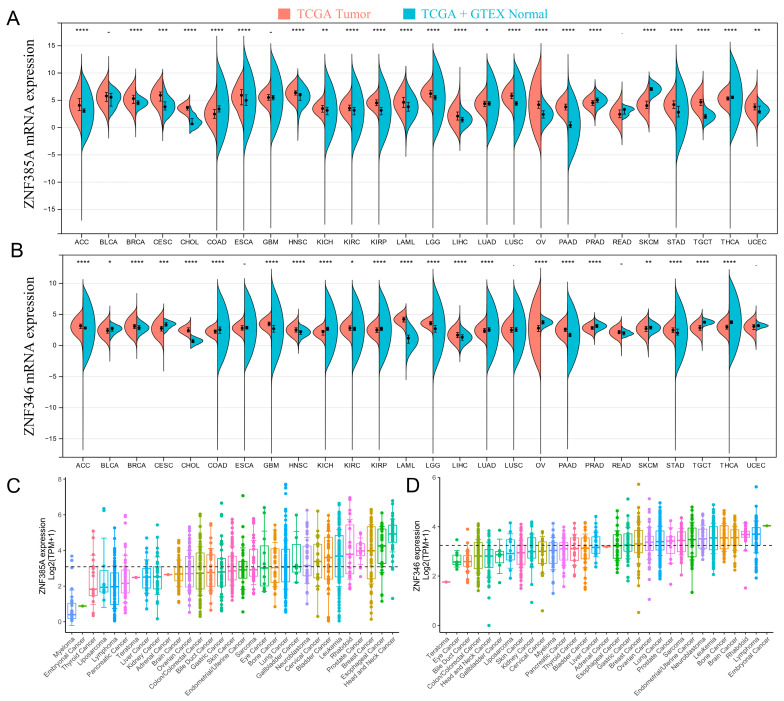
*ZNF385A* and *ZNF346* expression in different types of human cancers and cell lines. (**A**,**B**) Increased or decreased *ZNF385A* and *ZNF346* expression in different cancers, compared with normal tissues, by the TCGA and GTEx databases (* *p* < 0.05, ** *p* < 0.01, *** *p* < 0.001, **** *p* < 0.0001). (**C**,**D**) The expression of *ZNF385A* and *ZNF346* in tumor cell lines, based on the CCLE database.

We first investigated the pan-cancer expression patterns of *ZNF385A* and *ZNF346* via the Cancer Genome Atlas (TCGA) and Genotype Tissue Expression (GTEx) datasets. They were highly expressed in the majority of cancers, including ACC (Adrenocortical carcinoma), BRCA (Breast invasive carcinoma), CHOL (Cholangiocarcinoma), HNSC (Head and Neck squamous cell carcinoma), KIRC (Kidney renal clear cell carcinoma), LAML (Acute Myeloid Leukemia), LGG (Brain Lower Grade Glioma), LIHC (Liver hepatocellular carcinoma), PAAD (Pancreatic adenocarcinoma), and STAD (Stomach adenocarcinoma) (Figure 1A,B). *ZNF385A* and *ZNF346* were also ubiquitously expressed in a variety of cancer cell lines, based on the screening of expression data from the Cancer Cell Lineage Encyclopedia (CCLE) dataset (Figure 1C, D). After performing a pan-cancer overall survival analysis by GEPIA2, we observed that the high expression of *ZNF385A* was associated with poor prognosis in HNSC, KIRC, LAML, LIHC, THYM (Thymoma), and UVM (Uveal Melanoma), and a high expression of *ZNF346* was correlated with a poorer OS only in LIHC; therefore, we then focused on the analysis of *ZNF385A* and *ZNF346* on LIHC (Figure S1).

### 2.2. Expression Profiles of ZNF385A and ZNF346 in HCC

**Figure 2:**
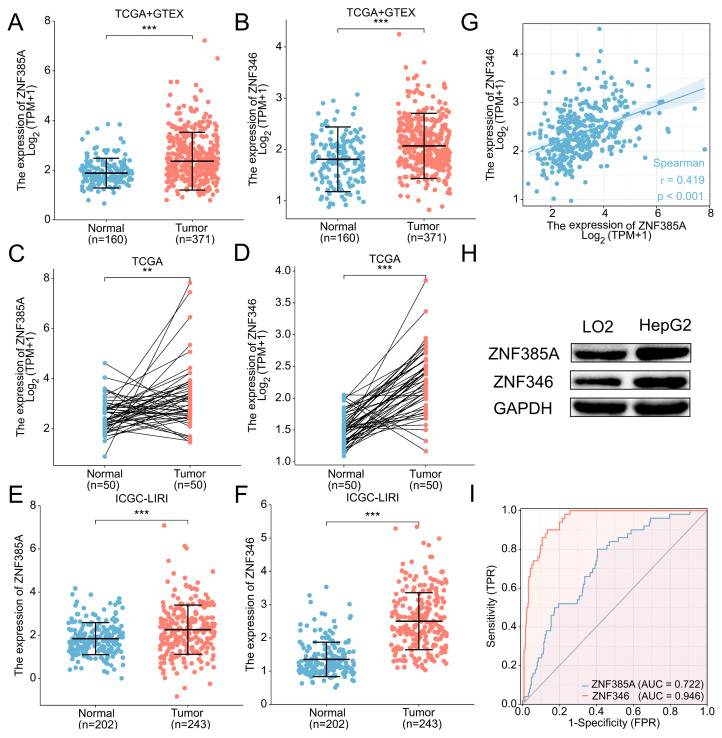
*ZNF385A* and *ZNF346* are overexpressed in HCC, and their diagnostic potential. (**A**,**B**) *ZNF385A* and *ZNF346* mRNA expression in 371 HCCs, and 160 adjacent normal tissues from the TCGA and GTEx databases. (**C**,**D**) *ZNF385A* and *ZNF346* mRNA expression in paired tumor and adjacent normal tissues of 50 patients with HCC in TCGA. (**E**,**F**) *ZNF385A* and *ZNF346* mRNA expression from the ICGC database. (**G**) Correlation scatter plot of *ZNF385A* and *ZNF346*. (**H**) Validation of ZNF385A and ZNF346 protein levels in normal liver cell (LO2) and HCC cell (HepG2) via Western blot analysis. (**I**) ROC curves to test the diagnostic value of *ZNF385A* and *ZNF346*. ** *p* < 0.01; *** *p* < 0.001.

The expression of *ZNF385A* and *ZNF346* in HCC tissues was found to be significantly higher in the cancerous tissues by evaluating the 531 samples (*p* < 0.001, Figure 2A,B) and the 50 paired tissues (*p* < 0.01, Figure 2C,D) in the TCGA database. The same trend was observed in the International Cancer Genome Consortium (ICGC) database (*p* < 0.001, Figure 2E,F). Interestingly, a significant positive correlation was found between *ZNF385A* and *ZNF346* (Figure 2G). In addition, we uncovered that the protein levels of ZNF385A and ZNF346 were remarkably upregulated in the HCC cell line (HepG2) with the normal liver cell line (LO2) as the control (Figure 2H). The results of the receiver operating characteristic (ROC) analysis showed that *ZNF385A* and *ZNF346* could be potential diagnostic biomarkers, with an area under the curve (AUC) of 0.722 and 0.946 in the TCGA cohort, respectively (Figure 2I). Furthermore, the clinical relevance analysis proved that the high expression of both *ZNF385A* and *ZNF346* was associated with HBV infection and higher alpha-fetoprotein (AFP) levels (Figure S2).

We next explored whether the distinct expression of *ZNF385A* and *ZNF346* in HCC was associated with genetic or epigenetic alterations. As presented in Figure S3, the mRNA expression of *ZNF385A* was significantly negatively correlated with its methylation levels but not *ZNF346*, and both of them were correlated with copy number variations (CNVs) in HCC. Since *ZNF385A* and *ZNF346* were rarely mutated in HCC (only one mutation in 353 samples), we analyzed oncogenic driver mutations in *ZNF385A* and *ZNF346* high and low expression groups. The results showed no significant difference in the overall mutation rates between the two groups. However, the *ZNF385A* and *ZNF346* high expression groups were enriched for the *p53* mutations (39.2% for *ZNF385A* and 52% for *ZNF346*), while the *CTNNB1* mutations were less frequent (24.8% for *ZNF385A* and 24.0% for *ZNF346*) (Figure S4). Finally, we hypothesized that *ZNF385A-* and *ZNF346*-high groups were enriched for the proliferation molecular subtype in HCC, which was characterized by high *p53* mutation rates, HBV infection, and high AFP levels [17].

### 2.3. The Prognostic Significance of ZNF385A and ZNF346 in HCC

**Figure 3:**
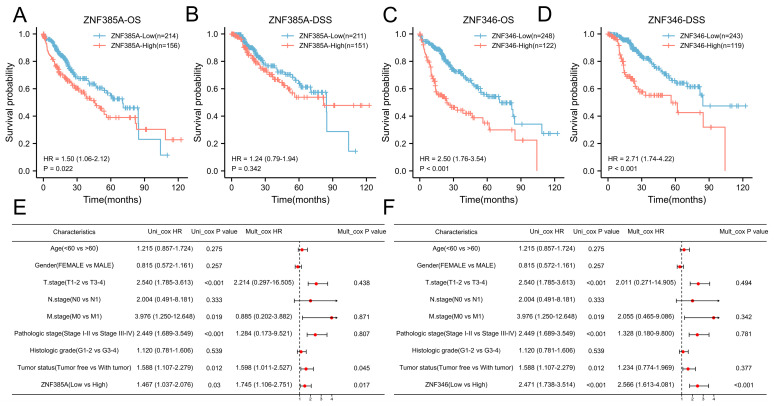
*ZNF385A* and *ZNF346* predict a poorer prognosis in HCC. (**A**–**D**) Survival curves of OS and DSS between high and low *ZNF385A*, and *ZNF346* expression subgroups in HCC. (**E**,**F**) Univariate and multivariate Cox regression analyses of *ZNF385A* (**E**) and *ZNF346* (**F**) for clinical factors.

By means of Kaplan–Meier survival analyses, high *ZNF346* expression was linked to poorer OS (*p* < 0.001) and disease-specific survival (DSS) (*p* < 0.001), while a higher expression of *ZNF385A* was related to worse OS (*p* = 0.022), but not the DSS (*p* = 0.342) (Figure 3A–D). After adjusting for several clinical parameters, *ZNF385A* and *ZNF346* were identified as independent risk factors for HCC by univariate and multivariate Cox regression analyses (Figure 3E,F). The survival analysis for the subgroups indicated that high *ZNF385A* and *ZNF346* expression both correlated with worse OS in HBV-infected subgroups compared with the non-infected subgroups (Figure S5), suggesting their possible involvement in the oncogenic process of HBV.

### 2.4. Biological Function and Pathway Analyses of ZNF385A and ZNF346 in HCC

**Figure 4:**
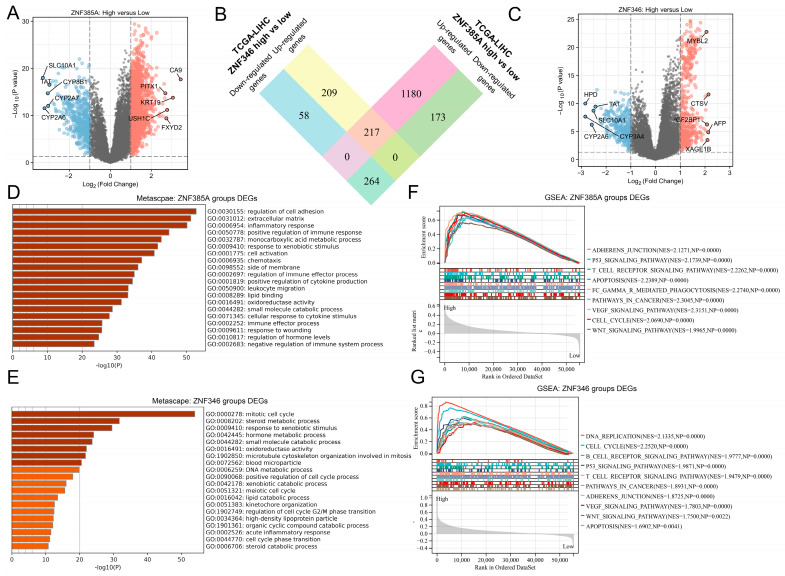
Function and pathway enrichment analyses of *ZNF385A* and *ZNF346* in HCC. (**A**) A volcano plot of *ZNF385A*-related DEGs in HCC, top 5 up- or down-regulated genes are visualized. (**B**) A Venn diagram showing *ZNF385A* and *ZNF346* intersecting DEGs (**C**) A volcano plot of *ZNF346*-related DEGs in HCC, top 5 up- or down-regulated genes are visualized. (**D**,**E**) GO enrichment analyses for *ZNF385A*- (**D**) and *ZNF346*- (**E**) related DEGs. (**F**,**G**) GSEA enrichment analyses in *ZNF385A* (**F**) and *ZNF346* (**G**) high expression groups.

To determine the underlying functions and mechanisms of *ZNF385A* and *ZNF346*, we calculated the differentially expressed genes (DEGs) between the *ZNF385A* and *ZNF346* high and low expression groups. A total of 1834 (Figure 4A) and 748 (Figure 4C) DEGs for *ZNF385A* and *ZNF346* were identified, respectively. Interestingly, there was a notable proportion of overlap between these DEGs (Figure 4B), which proved that *ZNF385A* and *ZNF346* may have certain commonalities in the carcinogenic mechanism in HCC. The *ZNF385A*- and *ZNF346*-related genes were further mapped into the Metascape database for gene ontology (GO) enrichment analysis. *ZNF385A* was mainly involved in cell adhesion and immune response (Figure 4D), and for *ZNF346*, cell cycle process was mainly enriched (Figure 4E). Both of them were notably associated with the inflammatory response. We further performed gene set enrichment (GSEA) analysis to explore signaling pathways that were markedly associated with *ZNF385A* and *ZNF346*. Many oncogenic signaling pathways of liver cancer were enriched in the *ZNF385A* and *ZNF346* high expression groups, which included the p53 signaling pathway, apoptosis, adherens junctions, the VEGF signaling pathway, the Wnt signaling pathway, and the cell cycle process (Figure 4F,G). Adherens junctions and the cell cycle are closely associated with cell migration and proliferation [18]. Furthermore, high expression groups showed the statistical enrichment of immune-related signaling pathways, such as T cell receptor signaling, B cell receptor signaling, and Fc gamma R-mediated phagocytosis pathways (Figure 4F,G). On the other hand, for the 217 intersecting up-regulated genes, 210 genes (97%) were risk factors for the unfavorable survival of HCC, of which 123 (57%) were statistically significant (Figure S6A). Similarly, for the 264 intersecting down-regulated genes, 258 genes (98%) were favorable for survival, of which 121 (46%) reached statistical significance (Figure S6B). Altogether, these provided evidence that *ZNF385A* and *ZNF346* may contribute to the malignant behaviors of HCC cells, and they were associated with immune and inflammation responses.

Until recently, the VEGF inhibitor sorafenib remained the only first-line treatment for advanced HCC. As the VEGF signaling pathway was enriched in the *ZNF385A*- and *ZNF346*-high groups, we predicted the chemotherapeutic response of sorafenib for each sample, based on the Genomics of Drug Sensitivity in the Cancer (GDSC) database. The results showed a lower IC_50_ value in the *ZNF385A* and *ZNF346*-high groups (*p* < 0.001) (Figure S6C,D), suggesting that they could be potential biomarkers for predicting a good therapeutic effect of sorafenib.

### 2.5. Knockdown of ZNF385A and ZNF346 Inhibits Proliferation and Migration in HCC

**Figure 5:**
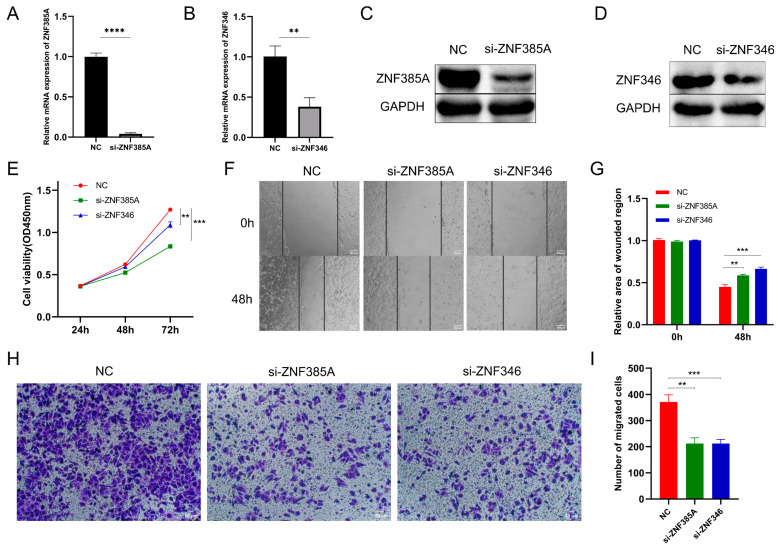
*ZNF385A* and *ZNF346* promotes cell proliferation and migration in HCC. (**A**,**B**) The effectiveness of si-*ZNF385A* (**A**) and *si-ZNF346* (**B**) in HepG2 cells via RT-qPCR. (**C**,**D**) The effectiveness of si-*ZNF385A* (**C**) and *si-ZNF346* (**D**) in HepG2 cells via Western blot. (**E**) Cell viability of different groups as measured using the CCK-8 assay. (**F**,**G**) Cell migration capacities of different groups as measured via wound healing assay. (**H**,**I**) Cell migration capacities of different groups measured via transwell assay. NC: cells transfected with negative control siRNAs that did not target any genes. ** *p* < 0.01, *** *p* < 0.001, **** *p* < 0.0001.

To validate the biological function of *ZNF385A* and *ZNF346* in HCC, we performed loss-of-function experiments by transfecting small interfering RNA (siRNA) into HepG2 cells. The effectiveness of *ZNF385A* and *ZNF346* knockdown was determined using quantitative reverse transcription PCR (RT-qPCR) and Western blot (Figure 5A–D). The cell counting kit-8 (CCK-8) assay demonstrated that a lower expression of *ZNF385A* and *ZNF346* drastically reduced the cell viability of HepG2 cells (*p* < 0.01) (Figure 5E). Then, the wound healing assay and transwell assay indicated that the knockdown of *ZNF385A* and *ZNF346* significantly inhibited the migration capacity (*p* < 0.01) (Figure 5F–I).

### 2.6. ZNF385A and ZNF346 Are Associated with an Inflamed Immunosuppressive TME in HCC

**Figure 6:**
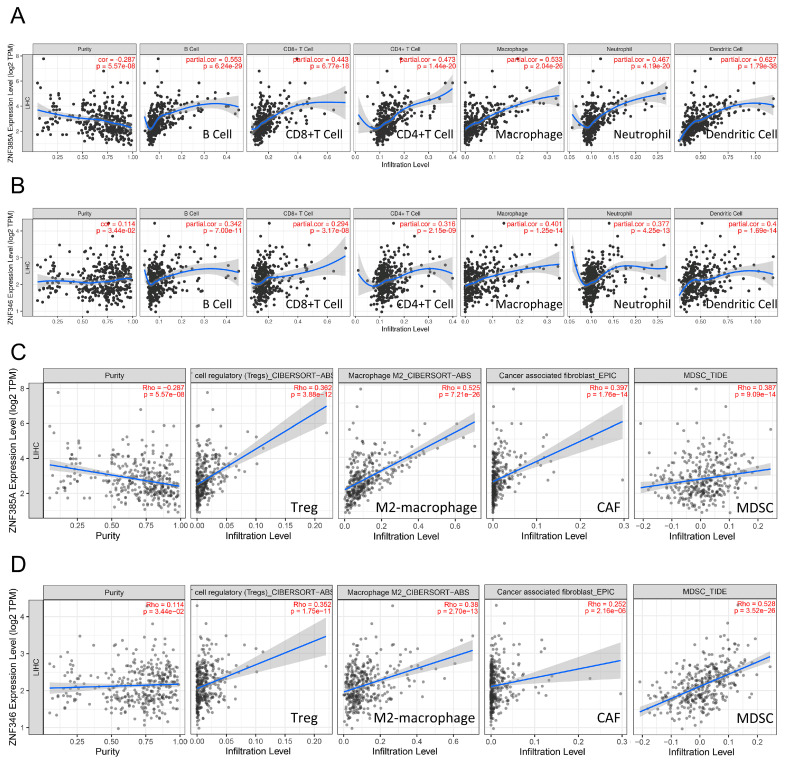
Correlation analyses between *ZNF385A* and *ZNF346* expression, and the immune infiltration of multiple immune cells. (**A**,**B**) Correlation with six main infiltrating cells in HCC via TIMER. (**C**,**D**) Correlation with four immunosuppressive cells in HCC via TIMER2.

Since the immune and inflammatory signaling pathways were noticeably enriched, we wondered if *ZNF385A* and *ZNF346* could modulate TME in HCC. According to the Tumor Immune Estimation Resource (TIMER) database, *ZNF385A* and *ZNF346* were significantly positively correlated with six main infiltrating immune cells in HCC, such as B cells, CD8 T cells, CD4 T cells, macrophages, dendritic cells, and neutrophils (Figure 6A,B). Next, we explored the immune landscape using different algorithms, and found that the *ZNF385A* and *ZNF346* level had a strong positive correlation with immunosuppressive cells including M2 macrophages, regulatory T cells (Tregs), myeloid-derived suppressor cells (MDSCs), and cancer-associated fibroblasts (CAFs) (Figure 6C,D), and most markers of them in HCC (Table 1). The homologous immune landscape in subgroups of *ZNF385A* and *ZNF346* was further verified by using two other independent algorithms: MCPcounter and quanTIseq (Figure S7).

**Figure 7:**
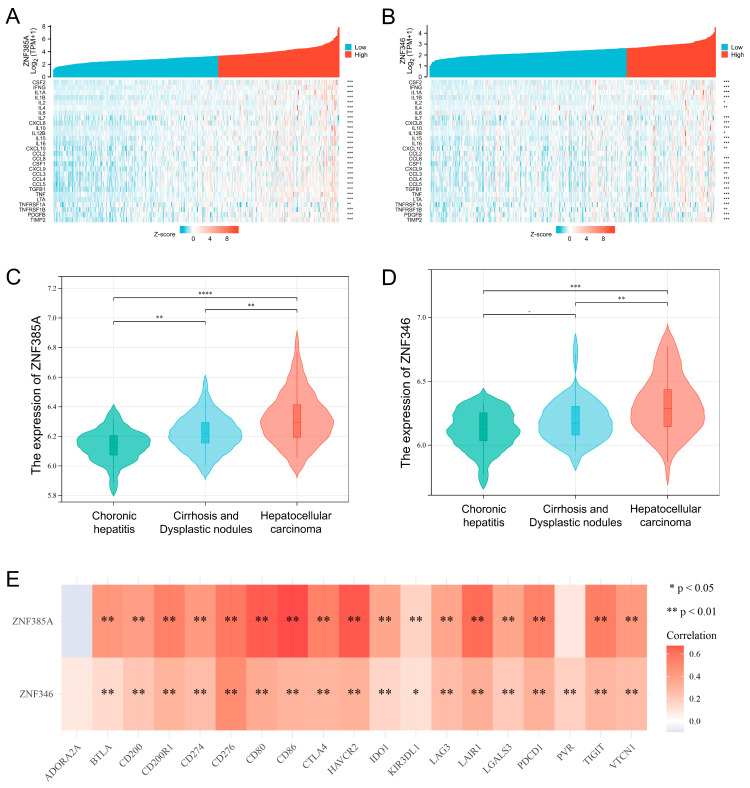
*ZNF385A* and *ZNF346* are associated with an inflamed TME in HCC. (**A**,**B**) Correlation analyses between *ZNF385A* (**A**), *ZNF346* (**B**) expression and inflammatory cytokines in HCC. (**C**,**D**) The *ZNF385A* (**C**) and *ZNF346* (**D**) expression status in different stages during HCC tumorigenesis based on data from GSE89377. (**E**) Correlation analyses between *ZNF385A*, *ZNF346* expression, and inhibitory immune checkpoints in HCC. * *p* < 0.05, ** *p* < 0.01, *** *p* < 0.001, **** *p* < 0.0001.

The analysis on the expression of inflammatory cytokines in the *ZNF385A* and *ZNF346* subgroups showed significantly higher levels of various inflammatory molecules in the *ZNF385A*- and *ZNF346*-high expression groups, including IL-1α, IL-1β, IL-10, and so on (Figure 7A,B). We downloaded the gene expression profiles of 94 samples covering different stages of HCC from GSE89377, found that the expression of *ZNF385A* and *ZNF346* gradually increased from chronic hepatitis to liver cirrhosis and HCC (Figure 7C,D). This result indicated that *ZNF385A* and *ZNF346* may participate in the inflammation response and liver carcinogenesis. Long-term exposure to chronic inflammation also leads to T-cell exhaustion by elevating the expression of checkpoint receptors [19]. It was identified that both *ZNF385A* and *ZNF346* were significantly and positively correlated with most inhibitory immune checkpoints, including *PD-1*, *PD-L1*, *IDO1*, *CTLA-4*, *TIGIT*, *LAG-3*, and *TIM-3* in HCC (Figure 7E). In combination with the above analysis, *ZNF385A* and *ZNF346* overexpression were associated with an immunosuppressive TME and chronic inflammation.

### 2.7. Overexpression of ZNF385A and ZNF346 Predict a Worse Efficacy of Immunotherapy

**Figure 8:**
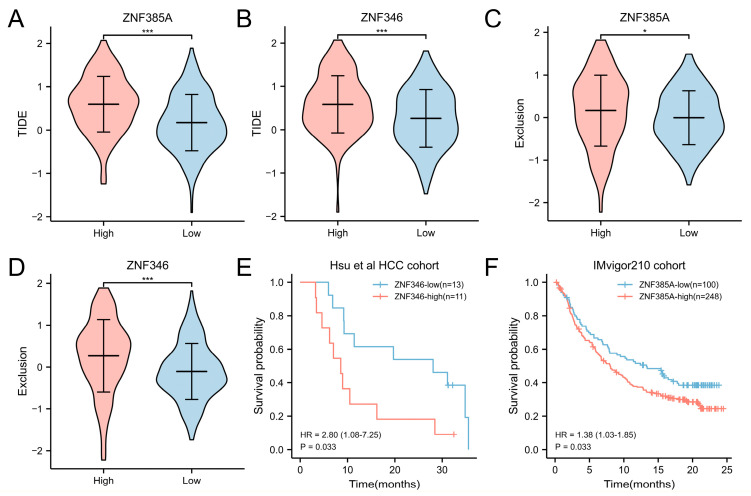
Overexpression of *ZNF385A* and *ZNF346* predicts resistance to immunotherapy. (**A**,**B**) The TIDE score in *ZNF385A* (**A**) and *ZNF346* (**B**) subgroups. (**C**,**D**) The T-cell exclusion score in *ZNF385A* (**C**) and *ZNF346* (**D**) subgroups. (**E**) Survival curves of the *ZNF346* subgroups in Hsu HCC cohort. (**F**) Survival curves of the *ZNF385A* subgroups in IMvigor210 cohort. * *p* < 0.05, *** *p* < 0.001.

Given the relevance of *ZNF385A* and *ZNF346* to an immunosuppressive TME, it was hypothesized that the high expression of *ZNF385A* and *ZNF346* may lead to immunotherapy resistance. We used the Tumor Immune Dysfunction and Exclusion (TIDE) score to assess the efficacy of immunotherapy in different *ZNF385A* and *ZNF346* subgroups. The results revealed that high *ZNF385A* and *ZNF346* expression groups exhibited higher TIDE (Figure 8A,B) and T-cell exclusion scores (Figure 8C,D), indicating poor immune checkpoint inhibitors (ICI) efficacy and a shorter survival time after ICI treatment. We then aimed to validate the prognostic significance of *ZNF385A* and *ZNF346* for immunotherapy. Hsu and colleagues tested 770 genes from 24 HCC patient samples with anti-PD-1/anti-PD-L1 treatment [20]. Within this cohort, the *ZNF346*-high group had worse OS than the *ZNF346*-low group, which was consistent with the TIDE prediction (Figure 8E). Since *ZNF385A* was not tested in Hsu HCC cohort, we introduced the IMvigor210 cohort (a trial investigating the clinical activity of anti-PD-L1 atezolizumab in metastatic urothelial carcinoma) [21]. Likewise, a high *ZNF385A* mRNA expression correlated with a poor survival prognosis (Figure 8F).

## 3. Discussion

*ZNF385A* and *ZNF346* both belong to RNA binding proteins, and they have very similar structures; however, there are very few studies in cancers. In the present study, pan-cancer analysis was conducted and a specific correlation analysis was focused on HCC. We found that the overexpression of *ZNF385A* and *ZNF346* were significantly correlated with a poor OS prognosis of HCC patients, and that it promoted the occurrence and development of HCC. In addition, they both exhibited a close relationship with tumor immunosuppression and inflammation, and they could predict the effect of immunotherapy in HCC.

The functional enrichment analysis of co-expressed genes and GSEA analysis indicated that *ZNF385A* and *ZNF346* were significantly related to cancer-related processes such as cell cycle, G2M checkpoint, DNA replication, and adherens junctions, and they were significantly enriched in cancer-related pathways such as p53, VEGF, and Wnt signaling pathways (Figure 4F,G). A recent study demonstrated that activated *p53* expression was positively correlated with the level of apoptosis and subsequent cancer development in chronic liver diseases [22]. The VEGF and Wnt signaling pathways have been widely reported to contribute to the carcinogenic process of HCC [23,24]. Here, we showed for the first time that the knockdown of *ZNF385A* and *ZNF346* in HepG2 cells resulted in a significant restraining effect on cell proliferation and migration (Figure 5). Therefore, the high expression of *ZNF385A* and *ZNF346* in HCC may be one of the reasons for the higher proliferative activity and metastatic ability of HCC cells.

The causes of liver cancer are complex, and viral infection leading to inflammation is one of the most common factors [25]. First of all, we noted that *ZNF385A* and *ZNF346* were more highly expressed and had higher hazard ratios (HRs) in HBV-infected HCC patients compared to those that were not infected with HBV (Figures S2 and S5), indicating that the two were along with the inflammatory process caused by HBV infection. Secondly, GO enrichment showed that *ZNF385A* and *ZNF346* co-expressed genes were significantly enriched in the inflammatory response, and GSEA analysis displayed that the high expression group was significantly enriched in the apoptosis pathway (Figure 4D–G). Similarly, previous studies illustrated that a large number of hepatocyte deaths usually lead to chronic inflammation [26,27], and that sustained increased apoptosis leads to regenerative proliferation and a high DNA replication rate [28]. Thirdly, more inflammatory cells, including lymphocytes, macrophages, and neutrophils, were infiltrated in the *ZNF385A* and *ZNF346* overexpression groups, and they were accompanied by many inflammatory cytokines that increased significantly (Figure 6A,B and Figure 7A,B). Dead hepatocytes are accompanied with the production of a great number of cytokines, chemokines, and growth factors, favoring an increase in the proliferation of HCC cells [29]. Finally, a dataset covering the different stages of hepatocellular carcinoma showed a trend of progressive increase in *ZNF385A* and *ZNF346* expression, from chronic hepatitis to hepatocellular carcinoma (Figure 7C,D). Hence, we hypothesized that *ZNF385A* and *ZNF346* may be involved in HBV-related inflammation during the progression of HCC.

Chronic unresolved inflammation can shape an immunosuppressive TME through multiple mechanisms [30]. We found *ZNF385A* and *ZNF346* overexpression was closely associated with the infiltration of immune-suppressive cells, including M2 macrophages, Tregs, MDSCs, and CAFs. M2 macrophages are the main inflammatory response cells that form an immunosuppressive microenvironment by secreting various growth factors, angiogenic factors, and cytokines [31]. Tregs are a subset of T cells that are characterized by significant immunosuppressive functions, and that are involved in the maintenance of immune homeostasis. In malignant conditions, Tregs can suppress the body’s anti-tumor immunity and promote tumor progression [32]. MDSCs are derived from bone marrow; they play a major role in suppressing anti-tumor immunity by impairing the function of effector T cells, as well as NK cells [33]. CAFs are key players in promoting liver tumor cell growth and enhancing their invasive ability [34]. Additionally, the overexpression of *ZNF385A* and *ZNF346* was associated with the upregulation of multiple inhibitory immune checkpoints (Figure 7E). These molecules lead to the loss of the anti-tumor function of T cells and can cause the immune escape of tumor cells [35]. The above findings implied that *ZNF385A* and *ZNF346* may promote the progression of HCC by influencing immune-suppressive cells.

Recently, immunotherapies have led to a paradigm shift in treatment for patients with HCC. However, despite the most effective therapy, dual VEGF/PD-L1 blockade doubled the response rates, and more than two-thirds of the patients still did not respond [36]. Therefore, it is critical to identify the patients who can benefit most from these therapies. By performing TIDE analyses and survival analyses in one liver cancer and one urothelial cancer cohort receiving anti-PD-1/anti-PD-L1 therapy, we observed that *ZNF385A* and *ZNF346* performed well in predicting the responses to ICI therapy (Figure 8).

Although this research provided multi-level evidence for the importance of *ZNF385A* and *ZNF346* in the development and progression of HCC, the main limitation of our study still existed. More experiments should be performed to validate the regulation of immunosuppressive cells by *ZNF385A* and *ZNF346*. In fact, our future research was ongoing to identify downstream target genes regulated by *ZNF385A* and *ZNF346*, that ultimately influence the TME and development of HCC. Taken together, we proposed *ZNF385A* and *ZNF346* as potential biomarkers and targets of HCC, and both of them were closely correlated with immunosuppressive cell infiltration and the inflammatory response.

## 4. Materials and Methods

### 4.1. Pan-Cancer Analysis of ZNF385A and ZNF346

The normalized and batch-corrected TPM expression values (UCSC Toil RNA-seq Recompute) were downloaded from the UCSC Xena project (https://xenabrowser.net/datapages/, accessed on 22 August 2022). A log2 (TPM + 0.001) transformation was applied to the expression values. The expression data of *ZNF385A* and *ZNF346* in cancer cell lines (version 22Q2) were downloaded from the DepMap portal (https://depmap.org/portal/, accessed on 4 September 2022). The pan-cancer survival map analysis was performed using GEPIA2 (http://gepia2.cancer-pku.cn/#index, accessed on 4 September 2022).

### 4.2. Expression and Survival Analyses of ZNF385A and ZNF346 in HCC

We downloaded the expression data of the TCGA and GTEx dataset from the UCSC Xena project, including 371 TCGA HCC tumor tissues, 50 TCGA paired normal tissues, and 110 GTEx normal tissues. The data were processed through Toil [37], and they were finally transformed to log2(TPM + 1) for further analysis. RNA sequencing expression profiles of LIRI-JP were obtained from the ICGC portal (https://dcc.icgc.org/releases/current/Projects, accessed on 15 September 2022). ROC curve analysis was completed using the “pROC” package in R [38]; the AUC of the ROC was calculated by integrating the area under the ROC curve.

The Kaplan–Meier survival analyses, and the univariate and multivariate Cox regression analyses were conducted using the “survival” and “survminer” package in R. The best cut-off value was determined using the “surv_cutpoint” function. According to the best cut-off value for OS, we defined the upper 42% (n = 156) expression level as *ZNF385A*-high, the upper 32% (n = 122) expression level as *ZNF346*-high, and the rest of the expression levels were defined as low expression groups, respectively.

### 4.3. Genetic Alteration Analysis

Correlations between *ZNF385A* and *ZNF346* expression, CNVs, and DNA methylation were retrieved from the cBioPortal platform (https://www.cbioportal.org/, accessed on 20 September 2022). For the mutation analysis, the top 24 mutational cancer driver genes in HCC were acquired from the Integrative Onco Genomics (IntOGen) database (https://www.intogen.org/search, accessed on 20 September 2022). The somatic mutation landscape in different groups was constructed utilizing the “maftools” package in R.

### 4.4. Screening of Co-Expressed Genes and Functional Enrichment Analysis

The identification of DEGs between the *ZNF385A* and *ZNF346* high and low expression groups was performed using the cBioportal platform. DEGs were visualized using volcano plots, with the criteria of absolute log (FC) > 1 and a *p*-value < 0.05. The intersection DEGs were extracted using the “VennDiagram” package in R. Metascape (https://metascape.org/gp/index.html, accessed on 3 October 2022) was applied for GO enrichment analysis. GSEA analysis was performed using GSEA software (version 4.2.3); we input the defined *ZNF385A* and *ZNF346* high and low expression groups into the software, and the gene set “c2.cp.kegg.v7.4.symbols.gmt” was selected. The number of permutations was 1000. Absolute NES (normalized enrichment score) >1.6 and *p*-value < 0.05 were set as cut-offs for significant enrichment. The chemotherapeutic response prediction was based on the “pRRophetic” package in R [39].

### 4.5. Immune Characteristics Analysis

TIMER (https://cistrome.shinyapps.io/timer/, accessed on 15 October 2022) was utilized to evaluate the correlation between *ZNF385A* and *ZNF346* expression, and six infiltrations of immune cells, including B cell, CD8+ T cell, CD4+ T cell, macrophage, neutrophil, and dendritic cell. Correlation analysis between *ZNF385A* and *ZNF346*, and four immunosuppressive cells (M2 macrophages, Tregs, MDSCs and CAFs), including their markers, were performed using the TIMER2 database (http://timer.cistrome.org/, accessed on 15 October 2022). The immune landscape in the *ZNF385A* and *ZNF346* subgroups was performed using the MCPcounter and quanTIseq algorithms, based on the “IOBR” package in R [40]. Correlations of *ZNF385A* and *ZNF346* with inflammatory cytokines and immune checkpoints were visualized using the “ggplot2” and “pheatmap” package in R.

### 4.6. Immunotherapy Response Prediction

The potential ICI response was predicted with the TIDE algorithm. TIDE is a computational method that is used to model two primary mechanisms of tumor immune evasion: the induction of T cell dysfunction in tumors with high cytotoxic T lymphocytes (CTL) levels, and the prevention of T cell infiltration in tumors with low CTL levels [41]. For immunotherapy datasets, the IMvigor210 was downloaded from http://research-pub.gene.com/IMvigor210CoreBiologies (accessed on 25 October 2022). The Hsu HCC cohort was downloaded from the Gene expression omnibus database (GEO, https://www.ncbi.nlm.nih.gov/geo/, accessed on 27 October 2022) with the accession number GSE140901.

### 4.7. Cell Culture and Transient Transfection

HepG2 cells and LO2 cells were donated by the Beijing Institute of Basic Medical Sciences; they were cultured in DMEM (Gibco, Thermo Fisher, Waltham, MA, USA) supplemented with 10% fetal bovine serum (FBS), and were placed in a 37 °C, 5% CO_2_ incubator. The transfection of HepG2 cell was performed, utilizing si-*ZNF385A*, si-*ZNF346*, or si-NC (GenePharma, Shanghai, China), using siRNA-Mate reagent (GenePharma, China) following the manufacturer’s recommendations. The sequences for si-*ZNF385A* were sense (5′–3′) GCCCGUCAUUUCCUGUAAUTT and antisense (5′–3′) AUUACAGGAAAUGACGGGCTT. The sequences for si-*ZNF346* were sense (5′–3′) GGGAAACGAAGAAGCUAGATT and antisense (5′–3′) UCUAGCUUCUUCGUUUCCCTT.

### 4.8. RT-qPCR

Total mRNA was extracted from cells 48 h after transfection using TRIzol solution (Thermo Fisher, Waltham, MA, USA). Reverse transcription and quantitative real time PCR (qPCR) were performed using PerfectStart Uni RT and qPCR kit (TransGen, Beijing, China). The qPCR assay was conducted on a CFX-96 system (Bio-Rad, Hercules, CA, USA); gene expression levels were normalized to the *GAPDH* gene using the 2^−∆∆Ct^ method. Primers for qPCR are listed in Table S1.

### 4.9. Western Blot Assay

Cells were lysed with cell lysis buffer (Beyotime, Shanghai, China) prior to BCA quantification (Beyotime, China), and 30 μg of total protein was loaded and separated with a 10% SDS-PAGE gel. Then, the protein was transferred onto PVDF membranes (Millipore, Burlington, MA, USA). After blocking with 5% skim milk, primary antibodies against ZNF385A (26288-1-AP) (1:2000, Proteintech, Rosemont, IL, USA), ZNF346 (20794-1-AP) (1:3000, Proteintech, Chicago, IL, USA), and GAPDH (10494-1-AP) (1:20,000, Proteintech, USA) were utilized to incubate the membranes throughout the night at 4 °C. The secondary antibody (A0208) (1:2000, Beyotime, China) was added the following day, and the protein bands were visualized with BeyoECL Plus (Beyotime, China).

### 4.10. Cell Proliferation and Migration Assays

The CCK-8 assay was performed to assess the proliferative capacity in different groups. A total of 3000 cells were seeded in each well of the 96-well plates. Then, 10 μL CCK-8 reagent (Beyotime, China) was added into each well at 24 h, 48 h, and 72 h post-transfection. After incubation at 37 °C for 1 h, the absorbance at 450 nm of each well was detected. The wound healing assay and transwell assay were performed to assess the migration capacity in different groups. For the wound healing assay, transfected cells were added to 24-well plates. Once the confluence reached 100%, a 200 μL pipette tip was used to scrape on the bottom of the culture plates. After removing the debris, the cells were incubated in serum-free medium for 24 h, then images were taken under an inverted microscope (Olympus, Tokyo, Japan). For the transwell assay, a 200 μL serum-free medium containing 5 × 10^4^ transfected cells was added into the upper chamber (Corning, New York, NY, USA), while a 600 μL medium that contained 10% FBS was introduced into the bottom chamber. After 24 h, the upper side of the chamber was thoroughly wiped off with cotton swabs, and the migrated cells were fixed with methanol and stained with 0.1% crystal violet. More than three fields were captured randomly under an inverted microscope (Olympus, Japan).

### 4.11. Statistical Analysis

Data were presented as mean ± standard deviation (SD) of at least three biological replicates. A standard Student’s *t*-test, paired *t*-test, or Mann–Whitney U-test was used to compare the differences within the two groups. For comparisons of more than two groups, ANOVA tests and Kruskal–Wallis tests were utilized. A correlation analysis was performed using Spearman’s correlation test. Cox regression was used for survival analyses. All statistical data analyses were implemented using R software (version 4.2.1) and GraphPad Prism 9.0.

## Figures and Tables

**Table 1:** Correlations between *ZNF385A*, *ZNF346*, and gene markers of immunosuppressive cells after adjusting for tumor purity via TIMER.

Cell Type	Gene Marker	*ZNF385A*		*ZNF346*	
		Correlation	*p*-Value	Correlation	*p*-Value
Treg	*FOXP3*	0.195	2.63 × 10^−4^	0.180	7.87 × 10^−4^
	*CCR8*	0.436	1.97 × 10^−17^	0.333	2.16 × 10^−10^
	*STAT5B*	0.257	1.27 × 10^−6^	0.376	4.83 × 10^−13^
	*TGFB*	0.631	1.10 × 10^−39^	0.311	3.67 × 10^−9^
CAF	*FAP*	0.378	3.58 × 10^−13^	0.260	9.76 × 10^−7^
	*PDGFRα*	0.274	2.35 × 10^−7^	0.123	2.25 × 10^−2^
	*PDGFRβ*	0.428	9.14 × 10^−17^	0.213	6.81 × 10^−5^
	*αSMA*	0.202	1.54 × 10^−4^	0.104	5.34 × 10^−2^
	*VIM*	0.505	1.10 × 10^−23^	0.297	1.93 × 10^−8^
MDSC	*CD11B*	0.49	2.97 × 10^−22^	0.391	4.82 × 10^−14^
	*CD33*	0.529	2.66 × 10^−26^	0.265	5.96 × 10^−7^
PMN-MDSC	*CD15*	0.365	2.73 × 10^−12^	0.350	2.20 × 10^−11^
M-MDSC	*CD14*	−0.378	3.65 × 10^−13^	−0.289	4.62 × 10^−8^
M2-macrophage	*CD115*	0.56	8.21 × 10^−30^	0.293	2.99 × 10^−8^
	*CD163*	0.342	7.08 × 10^−11^	0.191	3.60 × 10^−4^
	*CD206*	0.072	1.83 × 10^−1^	0.037	4.89 × 10^−1^
	*PPARG*	0.336	1.55 × 10^−10^	0.293	2.83 × 10^−8^
	*VSIG4*	0.366	2.26 × 10^−12^	0.204	1.34 × 10^−4^

Treg, regulatory T cell; CAF, cancer-associated fibroblast; MDSC, myeloid-derived suppressor cell; PMN-MDSC, polymorphonuclear myeloid-derived suppressor cell; M-MDSC, monocytic myeloid-derived suppressor cell.

## Data Availability

The publicly available data source and handling of these data are described in the Materials and Methods. More information is available from the corresponding author upon request.

## Supplementary Materials

The following supporting information can be downloaded at: https://www.mdpi.com/article/10.3390/ijms24043155/s1.

## References

[1] Sung H., Ferlay J., Siegel R.L., Laversanne M., Soerjomataram I., Jemal A., Bray F. (2021). Global Cancer Statistics 2020: GLOBOCAN Estimates of Incidence and Mortality Worldwide for 36 Cancers in 185 Countries. CA Cancer J. Clin..

[2] Siegel R.L., Miller K.D., Fuchs H.E., Jemal A. (2022). Cancer statistics, 2022. CA Cancer J. Clin..

[3] Llovet J.M., Kelley R.K., Villanueva A., Singal A.G., Pikarsky E., Roayaie S., Lencioni R., Koike K., Zucman-Rossi J., Finn R.S. (2021). Hepatocellular carcinoma. Nat. Rev. Dis. Primers.

[4] Chakraborty E., Sarkar D. (2022). Emerging Therapies for Hepatocellular Carcinoma (HCC). Cancers.

[5] Chiang C.L., Chan A.C.Y., Chiu K.W.H., Kong F.S. (2019). Combined Stereotactic Body Radiotherapy and Checkpoint Inhibition in Unresectable Hepatocellular Carcinoma: A Potential Synergistic Treatment Strategy. Front. Oncol..

[6] Finn R.S., Zhu A.X. (2021). Evolution of Systemic Therapy for Hepatocellular Carcinoma. Hepatology.

[7] Pinter M., Jain R.K., Duda D.G. (2021). The Current Landscape of Immune Checkpoint Blockade in Hepatocellular Carcinoma: A Review. JAMA Oncol..

[8] Binnewies M., Roberts E.W., Kersten K., Chan V., Fearon D.F., Merad M., Coussens L.M., Gabrilovich D.I., Ostrand-Rosenberg S., Hedrick C.C. (2018). Understanding the tumor immune microenvironment (TIME) for effective therapy. Nat. Med..

[9] Jen J., Wang Y.C. (2016). Zinc finger proteins in cancer progression. J. Biomed. Sci..

[10] Iuchi S. (2001). Three classes of C_2_H_2_ zinc finger proteins. Cell. Mol. Life Sci..

[11] Sharma S., Dimasi D., Higginson K., Della N.G. (2004). RZF, a zinc-finger protein in the photoreceptors of human retina. Gene.

[12] Sugimoto M., Gromley A., Sherr C.J. (2006). Hzf, a p53-responsive gene, regulates maintenance of the G2 phase checkpoint induced by DNA damage. Mol. Cell. Biol..

[13] Nakamura H., Kawagishi H., Watanabe A., Sugimoto K., Maruyama M., Sugimoto M. (2011). Cooperative role of the RNA-binding proteins Hzf and HuR in p53 activation. Mol. Cell. Biol..

[14] Yang M., Wu S., Su X., May W.S. (2006). JAZ mediates G1 cell-cycle arrest and apoptosis by positively regulating p53 transcriptional activity. Blood.

[15] Wei Q., Guo Z., Chen D., Jia X. (2020). MiR-542-3p Suppresses Neuroblastoma Cell Proliferation and Invasion by Downregulation of KDM1A and ZNF346. Open Life Sci..

[16] Wu T., Lin Y., Xie Z. (2018). MicroRNA-1247 inhibits cell proliferation by directly targeting ZNF346 in childhood neuroblastoma. Biol. Res..

[17] Villanueva A. (2019). Hepatocellular Carcinoma. N. Engl. J. Med..

[18] Gupta S., Yap A.S. (2021). How adherens junctions move cells during collective migration. Fac. Rev..

[19] Antonangeli F., Natalini A., Garassino M.C., Sica A., Santoni A., Di Rosa F. (2020). Regulation of PD-L1 Expression by NF-κB in Cancer. Front. Immunol..

[20] Hsu C.L., Ou D.L., Bai L.Y., Chen C.W., Lin L., Huang S.F., Cheng A.L., Jeng Y.M., Hsu C. (2021). Exploring Markers of Exhausted CD8 T Cells to Predict Response to Immune Checkpoint Inhibitor Therapy for Hepatocellular Carcinoma. Liver Cancer.

[21] Mariathasan S., Turley S.J., Nickles D., Castiglioni A., Yuen K., Wang Y., Kadel E.E., Koeppen H., Astarita J.L., Cubas R. (2018). TGFβ attenuates tumour response to PD-L1 blockade by contributing to exclusion of T cells. Nature.

[22] Makino Y., Hikita H., Fukumoto K., Sung J.H., Sakano Y., Murai K., Sakane S., Kodama T., Sakamori R., Kondo J. (2022). Constitutive Activation of the Tumor Suppressor p53 in Hepatocytes Paradoxically Promotes Non-Cell Autonomous Liver Carcinogenesis. Cancer Res..

[23] Perugorria M.J., Olaizola P., Labiano I., Esparza-Baquer A., Marzioni M., Marin J.J.G., Bujanda L., Banales J.M. (2019). Wnt-β-catenin signalling in liver development, health and disease. Nat. Rev. Gastroenterol. Hepatol..

[24] Morse M.A., Sun W., Kim R., He A.R., Abada P.B., Mynderse M., Finn R.S. (2019). The Role of Angiogenesis in Hepatocellular Carcinoma. Clin. Cancer Res..

[25] Tian Z., Xu C., Yang P., Lin Z., Wu W., Zhang W., Ding J., Ding R., Zhang X., Dou K. (2022). Molecular pathogenesis: Connections between viral hepatitis-induced and non-alcoholic steatohepatitis-induced hepatocellular carcinoma. Front. Immunol..

[26] Vucur M., Reisinger F., Gautheron J., Janssen J., Roderburg C., Cardenas D.V., Kreggenwinkel K., Koppe C., Hammerich L., Hakem R. (2013). RIP3 inhibits inflammatory hepatocarcinogenesis but promotes cholestasis by controlling caspase-8- and JNK-dependent compensatory cell proliferation. Cell Rep..

[27] Yang Y.M., Kim S.Y., Seki E. (2019). Inflammation and Liver Cancer: Molecular Mechanisms and Therapeutic Targets. Semin. Liver Dis..

[28] Boege Y., Malehmir M., Healy M.E., Bettermann K., Lorentzen A., Vucur M., Ahuja A.K., Böhm F., Mertens J.C., Shimizu Y. (2017). A Dual Role of Caspase-8 in Triggering and Sensing Proliferation-Associated DNA Damage, a Key Determinant of Liver Cancer Development. Cancer Cell.

[29] He G., Karin M. (2011). NF-κB and STAT3—Key players in liver inflammation and cancer. Cell Res..

[30] Huang H., Hu Y., Guo L., Wen Z. (2021). Integrated bioinformatics analyses of key genes involved in hepatocellular carcinoma immunosuppression. Oncol. Lett..

[31] Belgiovine C., D’Incalci M., Allavena P., Frapolli R. (2016). Tumor-associated macrophages and anti-tumor therapies: Complex links. Cell. Mol. Life Sci..

[32] Tanaka A., Sakaguchi S. (2017). Regulatory T cells in cancer immunotherapy. Cell Res..

[33] Gabrilovich D.I. (2017). Myeloid-Derived Suppressor Cells. Cancer Immunol. Res..

[34] Baglieri J., Brenner D.A., Kisseleva T. (2019). The Role of Fibrosis and Liver-Associated Fibroblasts in the Pathogenesis of Hepatocellular Carcinoma. Int. J. Mol. Sci..

[35] Kudo M. (2019). Scientific Rationale for Combination Immunotherapy of Hepatocellular Carcinoma with Anti-PD-1/PD-L1 and Anti-CTLA-4 Antibodies. Liver Cancer.

[36] Finn R.S., Qin S., Ikeda M., Galle P.R., Ducreux M., Kim T.Y., Kudo M., Breder V., Merle P., Kaseb A.O. (2020). Atezolizumab plus Bevacizumab in Unresectable Hepatocellular Carcinoma. N. Engl. J. Med..

[37] Vivian J., Rao A.A., Nothaft F.A., Ketchum C., Armstrong J., Novak A., Pfeil J., Narkizian J., Deran A.D., Musselman-Brown A. (2017). Toil enables reproducible, open source, big biomedical data analyses. Nat. Biotechnol..

[38] Robin X., Turck N., Hainard A., Tiberti N., Lisacek F., Sanchez J.C., Müller M. (2011). pROC: An open-source package for R and S+ to analyze and compare ROC curves. BMC Bioinform..

[39] Geeleher P., Cox N., Huang R.S. (2014). pRRophetic: An R package for prediction of clinical chemotherapeutic response from tumor gene expression levels. PLoS ONE.

[40] Zeng D., Ye Z., Shen R., Yu G., Wu J., Xiong Y., Zhou R., Qiu W., Huang N., Sun L. (2021). IOBR: Multi-Omics Immuno-Oncology Biological Research to Decode Tumor Microenvironment and Signatures. Front. Immunol..

[41] Jiang P., Gu S., Pan D., Fu J., Sahu A., Hu X., Li Z., Traugh N., Bu X., Li B. (2018). Signatures of T cell dysfunction and exclusion predict cancer immunotherapy response. Nat. Med..

