# Supplementary material for: ZNF385A and ZNF346 Serve as Prognostic Biomarkers Associated with an Inflamed Immunosuppressive Tumor Microenvironment in Hepatocellular Carcinoma
Source: Int J Mol Sci. 2023 Feb 5;24(4):3155. doi: 10.3390/ijms24043155 (PMC9962939; doi:10.3390/ijms24043155)
Supplement: Supplementary file 1 [file ijms-24-03155-s001.zip › ijms-2167707-supplementary.pdf]

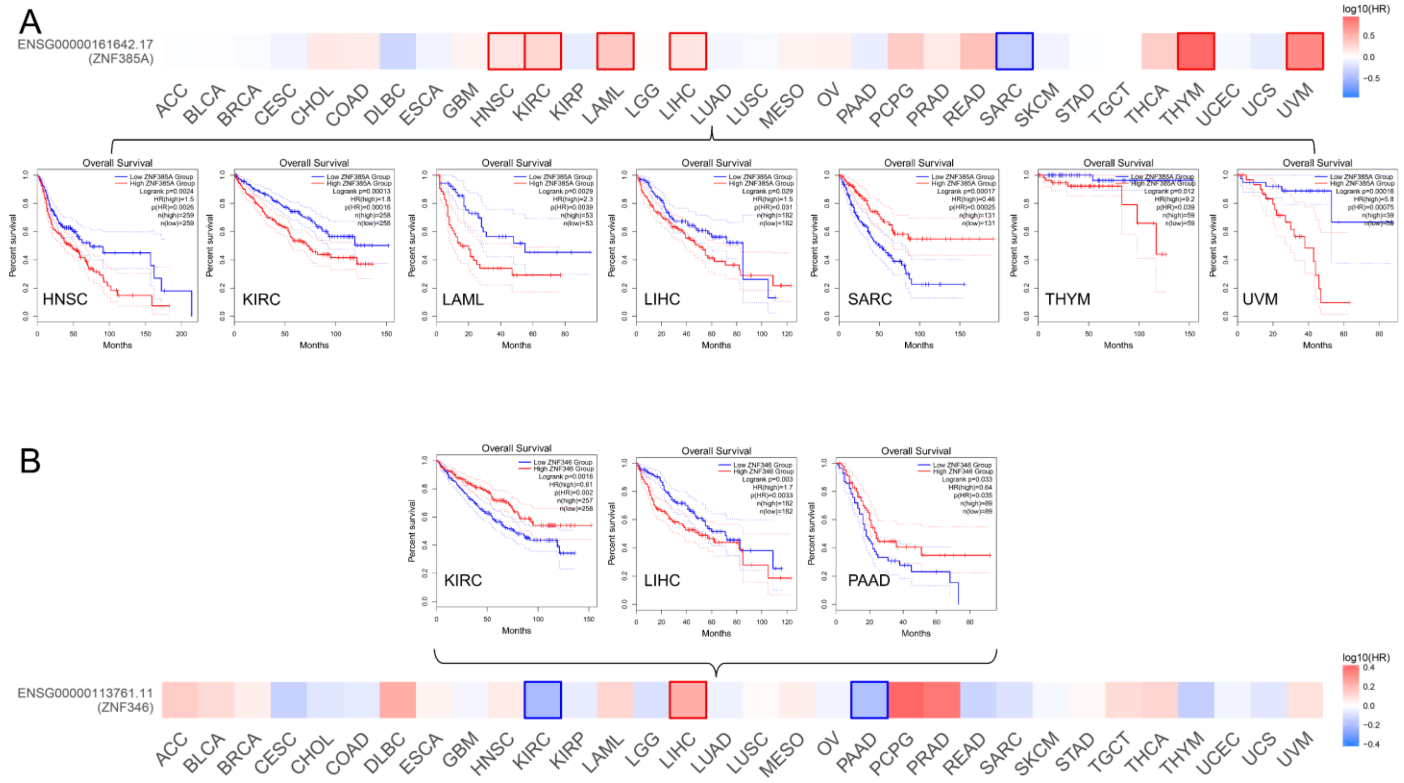

Figure S1. Correlation analysis between *ZNF385A* (A) and *ZNF346* (B) expression and prognosis of different cancers by GEPIA2. The survival map and Kaplan-Meier curves with positive results (highlighted by thickened borders in the survival map) were listed.

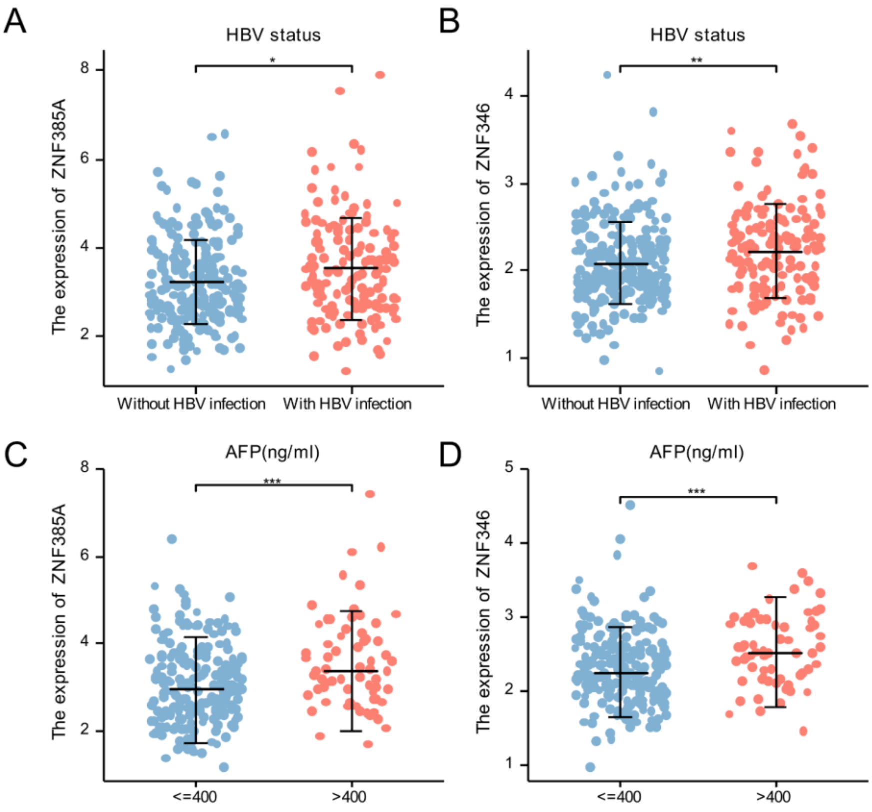

Figure S2. High *ZNF385A* and *ZNF346* expression both correlated with hepatitis viral infection and higher AFP levels (>400 ng/ml). * $p < 0.05$, ** $p < 0.01$, *** $p < 0.001$.

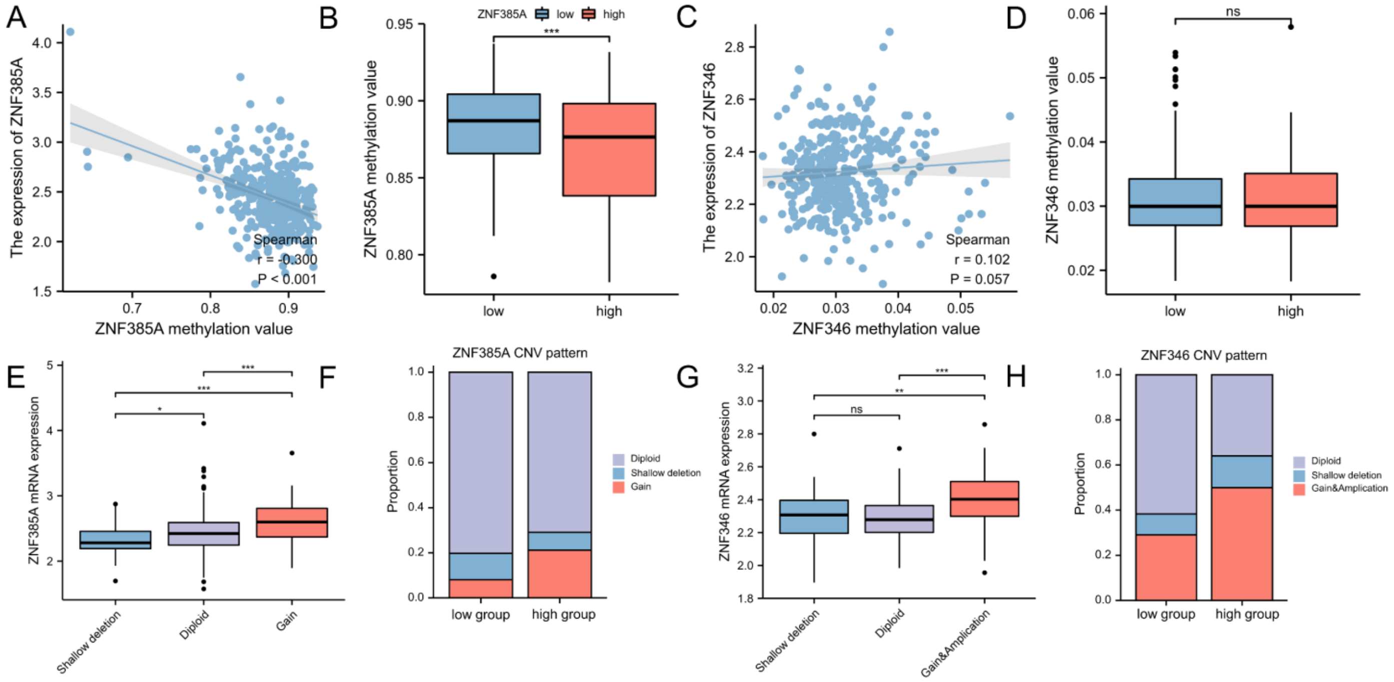

Figure S3. Epigenetic alteration features of *ZNF385A* and *ZNF346* in HCC. * $p < 0.05$, ** $p < 0.01$, *** $p < 0.001$.

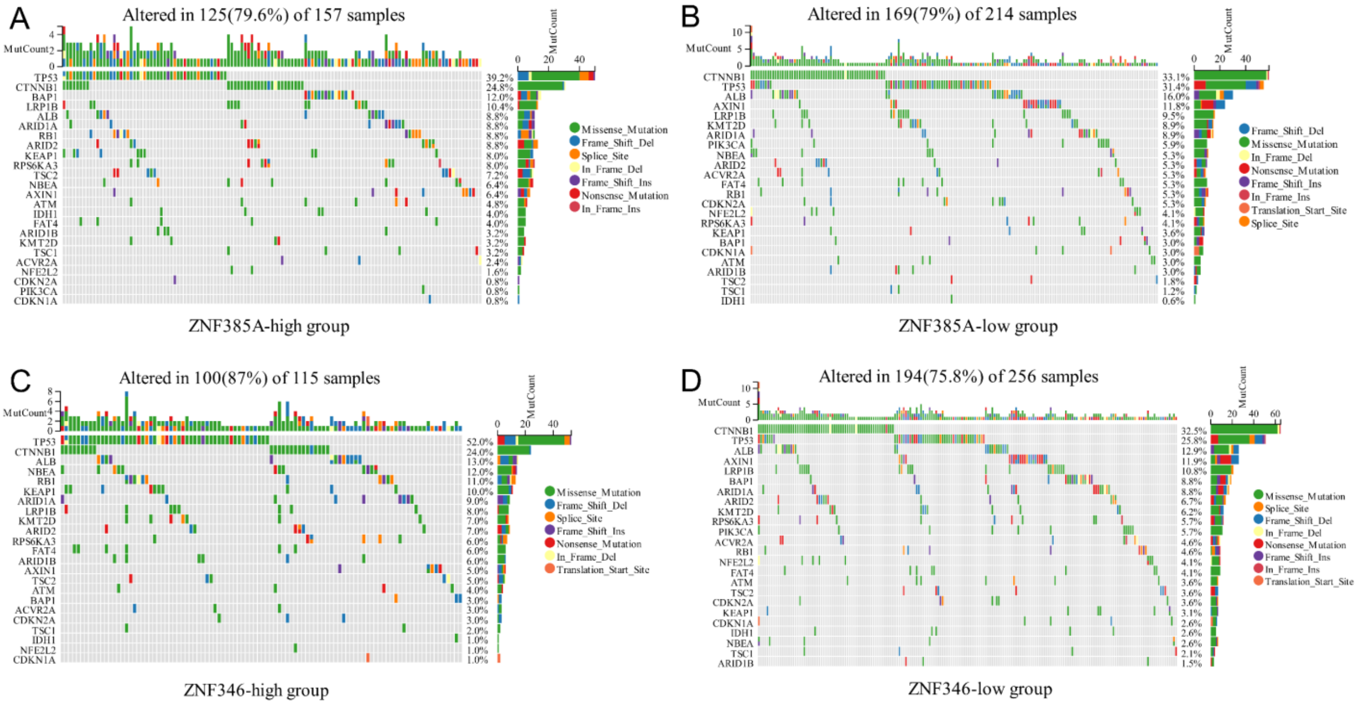

Figure S4. Genetic alteration profiles of driver genes in *ZNF385A* and *ZNF346* high and low expression groups in HCC.

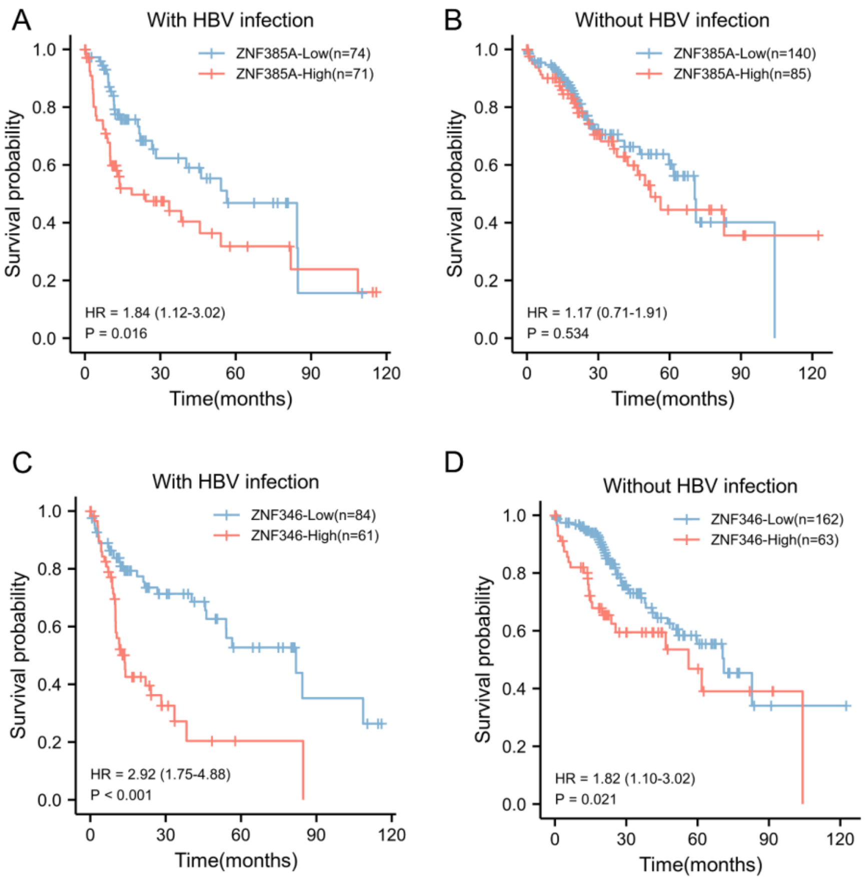

Figure S5. High *ZNF385A* and *ZNF346* expression in HBV-infected subgroups portended higher hazard ratio (HR).

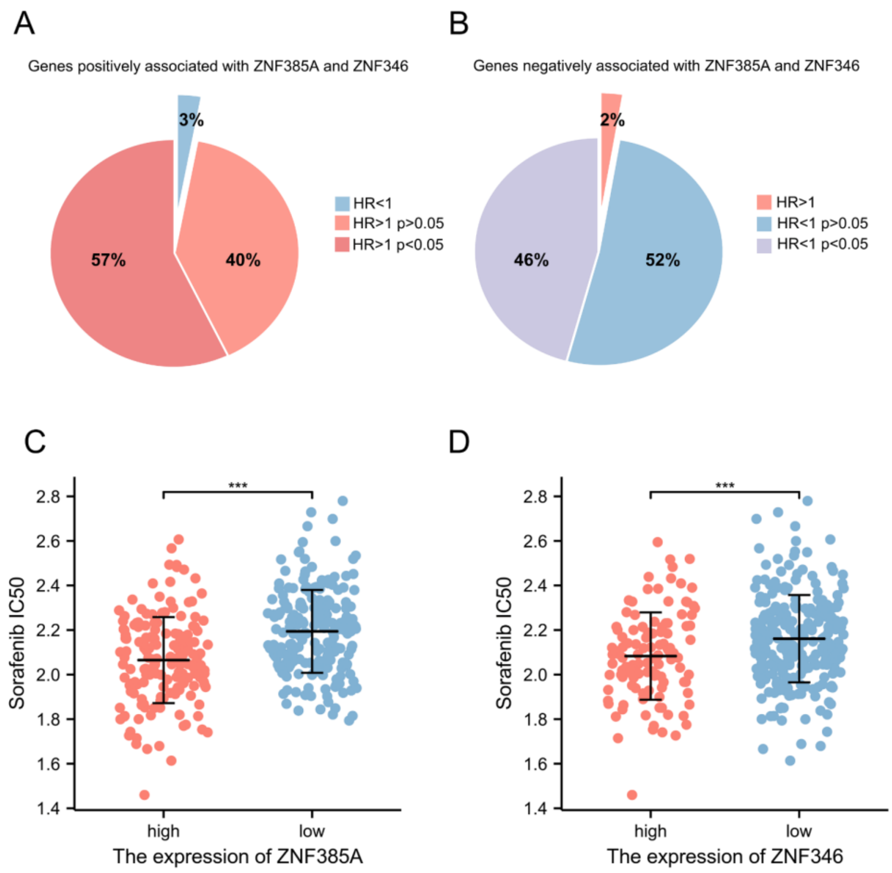

Figure S6. The prognosis of genes positively or negatively associated with *ZNF385A* and *ZNF346* and the chemotherapeutic response of Sorafenib in *ZNF385A* and *ZNF346* high and low expression groups. *** $p < 0.001$.

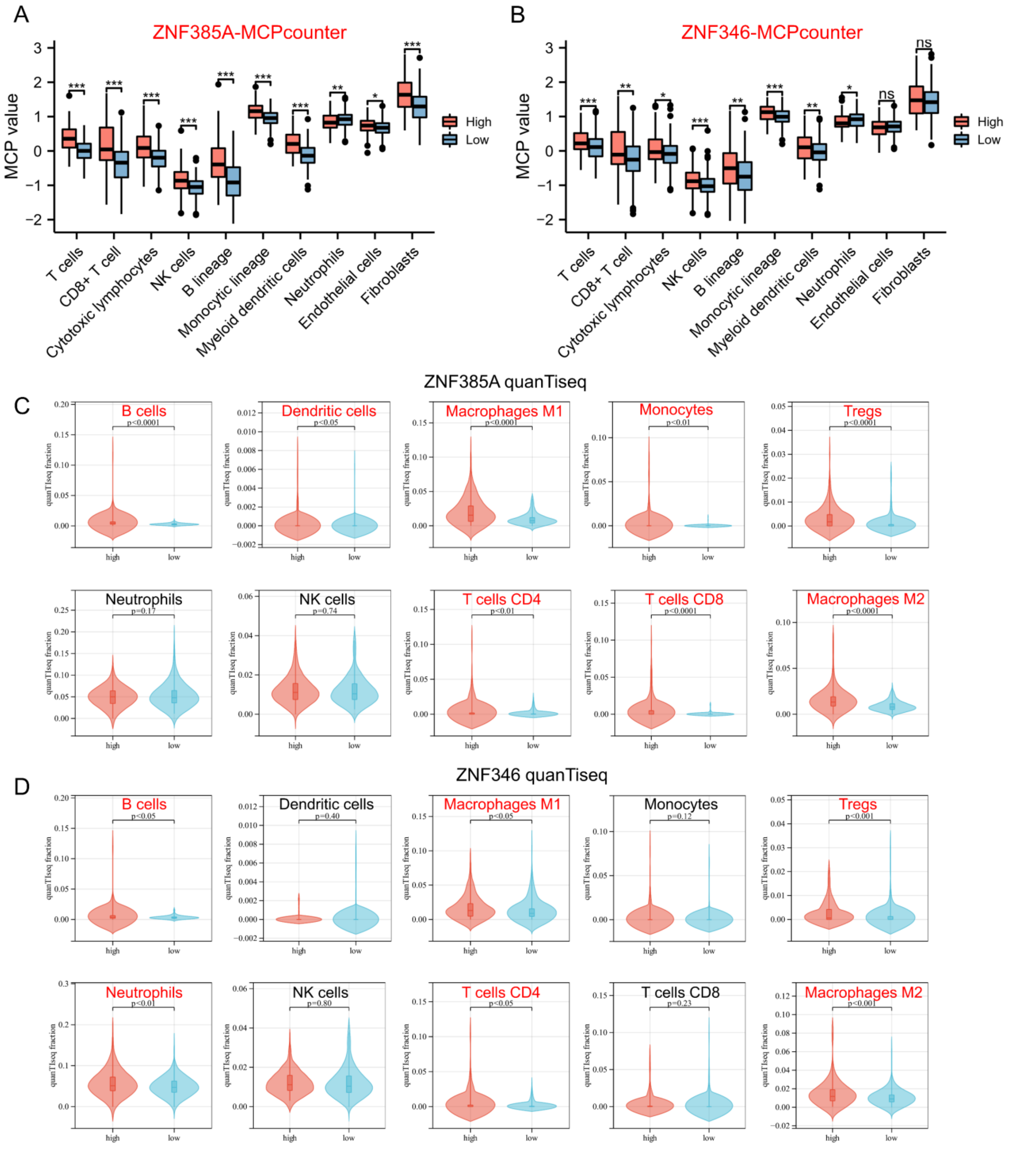

Figure S7. The immune landscape in *ZNF385A* and *ZNF346* high and low expression groups by MCPcounter and quanTiseq. * $p < 0.05$, ** $p < 0.01$, *** $p < 0.001$.

Table S1. Primers for qPCR

Gene	Forward 5′–3′	Reverse 5′–3′
ZNF385A	ACTGTGCTCTGTGCAAGGTG	GCTGGAGATGTGCTGTTTCA
ZNF346	CCTTACAGCAGCTCGGAGTTG	ACCTTACACTGGGTGTTGGTG
GAPDH	CGACCACTTTGTCAAGCTCA	AGGGGTCTACATGGCAACTG
